# Time-Frequency Analyses of Tide-Gauge Sensor Data

**DOI:** 10.3390/s110403939

**Published:** 2011-04-01

**Authors:** Serdar Erol

**Affiliations:** Department of Geomatics Engineering, Civil Engineering Faculty, Istanbul Technical University, Maslak 34469, Istanbul, Turkey; E-Mail: erol@itu.edu.tr; Tel.: +90-212-285-3826; Fax: +90-212-285-3414

**Keywords:** tide-gauge sensors, sea level, time series, spectral analysis, time-frequency analysis, LSSA, neural networks, wavelet transform, cross wavelet transform, wavelet coherence

## Abstract

The real world phenomena being observed by sensors are generally non-stationary in nature. The classical linear techniques for analysis and modeling natural time-series observations are inefficient and should be replaced by non-linear techniques of whose theoretical aspects and performances are varied. In this manner adopting the most appropriate technique and strategy is essential in evaluating sensors’ data. In this study, two different time-series analysis approaches, namely least squares spectral analysis (LSSA) and wavelet analysis (continuous wavelet transform, cross wavelet transform and wavelet coherence algorithms as extensions of wavelet analysis), are applied to sea-level observations recorded by tide-gauge sensors, and the advantages and drawbacks of these methods are reviewed. The analyses were carried out using sea-level observations recorded at the Antalya-II and Erdek tide-gauge stations of the Turkish National Sea-Level Monitoring System. In the analyses, the useful information hidden in the noisy signals was detected, and the common features between the two sea-level time series were clarified. The tide-gauge records have data gaps in time because of issues such as instrumental shortcomings and power outages. Concerning the difficulties of the time-frequency analysis of data with voids, the sea-level observations were preprocessed, and the missing parts were predicted using the neural network method prior to the analysis. In conclusion the merits and limitations of the techniques in evaluating non-stationary observations by means of tide-gauge sensors records were documented and an analysis strategy for the sequential sensors observations was presented.

## 1. Introduction

The surface of the sea deforms continuously. Its level, measured relative to an arbitrary datum, is called ‘sea level’ and changes with time and is the most obvious indicator of ocean changes. Changes in sea level are greater in the shallow waters near a coast than in the open sea, and, because a large fraction of the human population resides in coastal areas, variations in sea level have aroused interest for a long time. Knowledge of the near-shore sea-level variations is of great importance for safe navigation, and sea-level observations provide valuable input to ocean science and to geodynamic and geoscience applications [1]. With these goals, sea-level data from tide gauges all over the world are archived and distributed by an international service, the Permanent Service for Mean Sea Level (PSMSL) [2] along with a history of the datum with respect to which the sea level was measured. As a member of this service, the Turkish National Sea-Level Monitoring System (TUSELS and its Turkish abbreviation is TUDES) provides data to PSMSL.

Time-series analysis is a fundamental issue in evaluating sea-level observations and identifying the tidal components of sea-level changes, as in many other fields of empirical research [3,4]. Considering tide-gauge sensor records, one almost always faces a composite of numerous scales ranging from days to decades. On the considered time scales, sea-level changes are often non-stationary and time resolved methods are necessary for an insightful analysis of the data [5]. In the spectral analyses of sea-level variations, filtering the tides and other high-frequency oscillations out of the observations is required to obtain the seasonal sea-level cycle. This filtration is most easily achieved by averaging the hourly sea level over a month to obtain a ‘monthly sea level’. Frequently, the sea-level records contain gaps and irregular sampling intervals originating from failures in the measuring/recording equipment or the upgrade of a tide gauge. These gaps introduce difficulties and uncertainties into the stages of data analysis and prediction. Therefore, either using a suitable method of analysis, which can evaluate unequally spaced, gappy data, or preprocessing the data to fill the missing data using an appropriate prediction algorithm is required. In this study, we aim to provide a methodological review for time-frequency analysis of non-stationary sensors observations using the least squares spectral analysis (LSSA) and wavelet analysis separately and clarifying superiorities and weaknesses of the experimented techniques. With this purpose we applied the techniques to estimate the spectra of the sea-level changes, employing the 19-year and 10-year data recorded at the Antalya-II and Erdek tide-gauge stations, respectively. The missing parts of the data were predicted using the neural network (NN) method.

LSSA is a least squares estimation method for computing variance- and power-spectra and suggested by [6,7] as an alternative to classical Fourier spectral analysis (see, e.g., [8]). In this method, the optimization in the Euclidean sense offers numerous advantages over using the other classical spectral evaluation methods. Its most important advantage is that time series with unequally spaced values and gaps can be analyzed without preprocessing, which may corrupt or obligate useful information hidden in the series [9–11]. It has been applied in its original [7] or alternative forms by a number of researchers in many fields, such as geodetic science (e.g., [9,12–20]) and observational astronomy (e.g., [10,21,22]).

Wavelet analysis is another method that can be used to analyze time series that contain non-stationary powers at many different frequencies [23,24]. Recently, the wavelet-analysis method has become a common way of analyzing localized power variations within a time series in various disciplines and applications such as climatology, atmospheric science and geoscience [25–34]. By decomposing a time series into a time-frequency space, the dominant modes of variability and the variation of those modes with time can be determined. Wavelet analysis overcomes the limitations of classical frequency-space analysis methods that assume that the underlying processes are stationary in time. There are two classes of wavelet analysis: the continuous wavelet transfrom (CWT) and its discrete counterpart. The discrete wavelet transform is a compact representation of the data and is particularly useful for noise reduction and data compression whereas the CWT is better for intuitive feature extraction purposes. When investigating the sea-level data, we are typically interested in extracting low signal-to-noise-ratio signals in the time series and apply CWT to the data. However, because analyzing the data using CWT requires equally spaced values, the gaps in the used data sets were filled using the multi-layer feedforward backpropagation neural network (MLFB-NN) method before the data analysis. The neural-network method can satisfactorily represent any arbitrary nonlinear function when a properly trained neural network is used. With this method, useful relationships among different inputs and outputs can be clarified. The MLFB algorithm is commonly used for training the neural networks in many applications. The performance of this algorithm is reported to be satisfactory in the prediction of the values in time series [18,35–38].

Although CWT is a common tool for analyzing localized intermittent oscillations in time series, it is very often desirable to examine together two time series that are expected to be linked in some way. In particular, it may be useful to examine whether regions in time-frequency space with large common power have a consistent phase relationship and therefore are suggestive of causality between the time series [30]. From the CWTs of Erdek and Antalya-II tide-gauge records, we constructed the cross wavelet transform (XWT) which exposes the common power and relative phase of two sea-level data sets in time-frequency space, thus revealing the differences and similarities of the sea-level changes recorded in the open and semi-enclosed seas with respect to the locations of the Antalya-II and Erdek tide gauges. Another useful quantity in measuring the cross-correlation between two time series as a function of frequency is the wavelet coherence (WTC). WTC is defined as the square of the cross-spectrum normalized by the individual power spectra, and it allows the determination of high levels of significance even when the common power of the two series is low. For this reason, this wavelet tool has been called “an accurate representation of the (normalized) covariance between the two time series” by [27,33]. Similar to XWT, we also generated the WTC of the two time series to inspect their common powers and the phase difference and compared the results from both wavelet tools.

The results of this study confirmed the applicability of the employed techniques in analyzing and investigating the sea-level variations recorded by tide-gauge sensors. The LSSA is a very useful technique in spectral analysis for inspecting and clarifying periodic signals hidden in noisy time series with trends. In the prediction of the missing data in sea-level series, the neural-network method worked well, considering the quality measures of the prediction. Because natural series, like sea-level observations, are generally non-stationary, the ability of neural networks to model non-linear processes without any a-priori assumptions about the generating processes provides an advantage in prediction. The significant periodicities revealed by LSSA were confirmed in the results of the wavelet analysis. Furthermore, the correlation between the time series of the two tide gauges was explained using the wavelet tools. Wavelet is a strong method for the time-frequency analysis of non-stationary sequential data and is suggested for investigating sea-level changes.

## 2. Tide-Gauge Stations in Turkey

**Figure 1.**
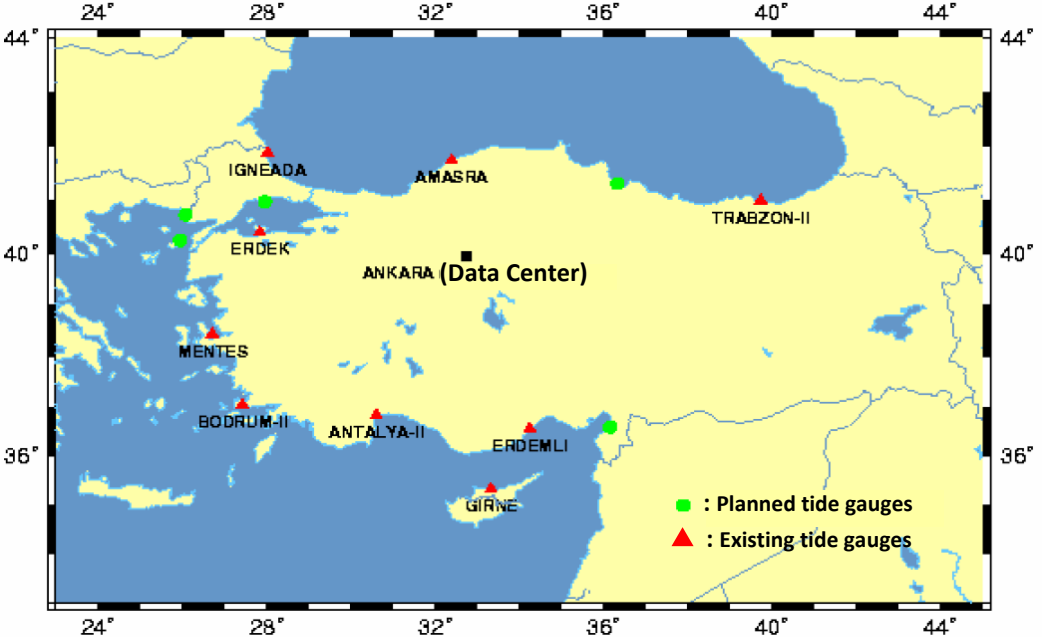
TUSELS tide-gauge stations in Turkey [42].

TUSELS presently consists of a data center in Ankara and a series of operational tide gauges located along the surrounding Mediterranean-, Marmara-, Aegean- and Black-Sea coasts of Turkey (see Figure 1 for the current structure of TUSELS with active and planned tide gauges on the Turkish coast) [39–42].

**Figure 2.**
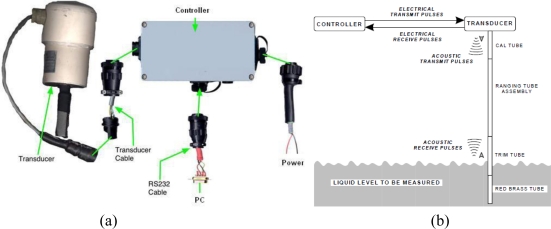
**(a)** the units of the digital acoustic tide-gauge sensor with cable connections **(b)** the illustration of the measurement system with acoustic tide-gauge sensor [44].

Sea-level monitoring studies in Turkey began in 1930s, and the General Command of Mapping (GCM) has the responsibility of establishing and operating the TUSELS tide gauges and distributing their data. The activities of transferring, quality control and analysis of tide gauge-data are carried out at the data center in Ankara. In 1998 and 1999, the tide gauges were modernized and all existing analogous floating type tide-gauge sensors in stilling wells were upgraded to digital and automatic devices by GCM in order to meet the GLOSS (Global Sea-level Observing System) standards [43]. Today, the stations are equipped with a measurement and data-collection unit with self-calibrating acoustic-ranging sea-level sensors (Aquatrak 4100 series) and meteorological sensors. Figure 2 shows the units of the sea-level sensor with its cable connections and an illustration of the sea-level measurement principles using the acoustic sensor [44]. The measurement principle of the Aquatrak sensor is as follows: a series of electrical pulses are transmitted from the controller unit to the transducer that converts them into acoustic pulses and sends them to the sea surface via a sounding tube. The sounding tube is a collective name for a calibration (Cal), ranging, trim and red-brass tube that each has a different function in transmitting the acoustic pulse. As the acoustic signal passes down through the tube an echo is produced that is returned to the controller receiver. When the pulse strikes the liquid surface another echo is produced, which is also returned to receiver. A special technique is based upon the comparison of a pulse time of travel within the known (through the calibration tube) to an unknown distance to the liquid level (through the sounding tube). The controller initiates the drive pulse to the transducer, times and stores the calibration return echo and times and stores the liquid level return echo. An on-board microprocessor calculates the ratio, applies the offset values, performs the unit conversions and stores the data then transmits the sea-level value to the data logger (or PC) in directly readable ASCII units [44].

**Figure 3.**
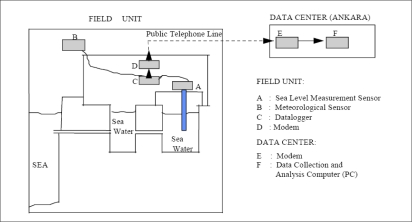
The structure of a digital TUSELS tide-gauge station [39].

After modernization the tide-gauge stations, [42] reports that the datum connection between the analog and the digital and automatic sea-level measurement systems was achieved by first-order precise leveling and the datum of the new system (acoustic sea-level measurement device) being transformed to the old system’s datum (analog floating sea-level measurement system with stilling well), thus providing data continuity of sea-level measurements. The hourly sea-level values for 18 years (1985–2003) of Antalya-II and 19 years (1984–2003) of Erdek tide gauges have been quality controlled by comparing them with the predicted values after removal of the datum shifts and time errors. Today, the hourly and daily sensor data of tide gauges are transferred to and analyzed in the Data Center (Figure 3 shows the structure of a modernized digital tide gauge of TUSELS and the data flow chart) [39,42]. Daily values are computed by applying a 119-point low-pass filter to the hourly sea-level values, and monthly values are obtained from the daily values with a simple averaging and released to the users and researchers via the global data bank of PSMSL [2].

**Figure 4.**
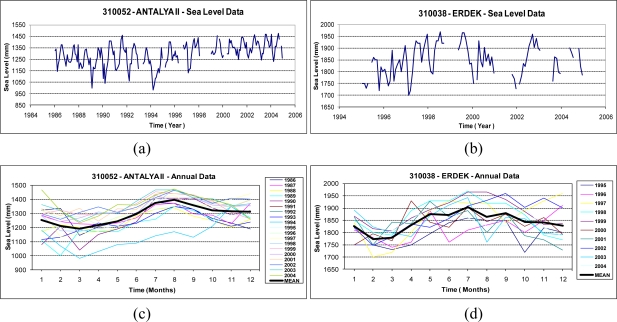
The sea-level data of Antalya-II and Erdek tide gauges for the considered time span: **(a, b)** the entire data span, **(c, d)** the annual changes of sea level and their mean.

**Figure 5.**
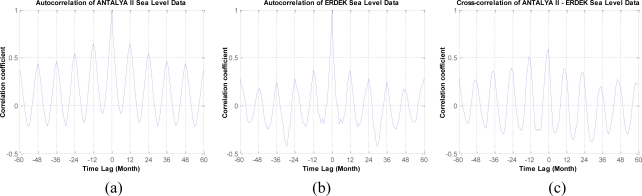
Plots of the autocorrelation functions of the sea-level observations for the **(a)** Antalya-II and **(b)** Erdek tide gauges and **(c)** the cross-correlation function between the time series (sea-level observations) of the Antalya-II and Erdek tide-gauges records.

In the numerical tests in this investigation, the monthly data from the Antalya-II and Erdek tide gauges, downloaded from [2], were analyzed. The Erdek tide gauge was installed on the Marmara coast of Turkey (see Figure 1) at the end of 1984 and operated using analog sensors until its upgrade in April 1999. Now, its digital and automatic sensors are capable of providing high-quality sea-level data. The Antalya-II tide gauge is on the Mediterranean coast of Turkey (see Figure 1) and was activated in 1985. Similar to the Erdek one, the Antalya-II tide gauge operated using the analog system until 1998, at which point its system was also upgraded with acoustic sensors [42]. The Antalya-II tide-gauge station has special importance as being the official zero-point of the Turkish National Vertical Datum. The specifications of both tide gauges are summarized in Table 1. The data used in this work span the years of 1986–2005 for Antalya-II and 1995–2005 for Erdek. The specified data intervals, considered in the analyses were determined by the data availability of the PSMSL data bank at the date of this study. The graphics in Figure 4 shows the monthly sea-level observations considered in the time-frequency analysis, and the autocorrelation and cross-correlation graphs of the tide-gauge records are shown in Figure 5.

The autocorrelation functions of sea-level observations recorded at Antalya-II and Erdek (see Figure 5(a,b)) reveal the presence of a periodicity. When the correlograms in the figures are considered, it is seen that the autocorrelation coefficient has peaks every 12 months—a time lag. Therefore, the highest period can be assumed to be 12 months for each time series. The graph of the cross-correlation functions between the time series of both tide-gauge sensors is shown in Figure 5(c). In this correlogram, the correlation between the sea-level signals at Antalya-II and Erdek with a 12-month time lag is seen. The correlation functions verify the existence of a periodicity in the time series; however, the following methods provide a more rigorous investigation of the sea-level changes in the period.

In the results of the GCM’s harmonic analysis [45] of the 1984–2003 monthly sea-level data from the tide gauges, the relative mean sea-level changes at Antalya-II and Erdek are reported to be 8.7 ± 0.8 mm/yr and 9.6 ± 0.9 mm/yr, respectively, and these values are reported to be much higher than the global sea-level rise estimates [42]. An investigation of these relative sea-level rises against episodic GPS observations and the precise leveling measurements revealed significant vertical-movement rates of −5.3 ± 1.8 mm/yr and −8.4 ± 3.0 mm/yr for Antalya-II and Erdek, respectively. Based on these findings, the relative sea-level changes at Antalya-II and Erdek tide gauges are purported to be caused by the local or regional subsidence of the crust in which those tide gauges are located [42]. These results by [42] emphasize the importance of studies investigating and clarifying the sea-level trends and periodicities for human life and future planning in the coastal areas of Turkey.

## 3. Time-Series Analysis

A set of observations or results obtained from a physical process, arranged in a specific manner, is called a data series. If the data series has a chronological ordering, it constitutes a time series [20]. There are two basic approaches to analyzing time series: the time domain and the frequency domain. In time-domain analysis, the relationship of an observation at time *t* to the observations at previous time points is examined and modeled. In the frequency-domain approach, the sinusoidal components across the series are examined using spectral analysis. The time series can be characterized equivalently in terms of the auto-covariance function in the time-domain or in terms of the spectral-density function in the frequency domain.

Spectral analysis techniques permit the identification of periodicities or hystereses in the time-series and their decomposition into periodic signals. In the cases of measurements of small amplitudes and high noise-to-signal ratios, reflecting the superposition of different signals, spectral-analysis techniques provide the best results [16,46]. Using a special algorithm, least squares spectral analysis, even unequally sampled and gappy data, such as the sea-level time series, can be analyzed. The appropriate analysis of the time series of the sea-level observations with mathematical and statistical methods will clarify the magnitude and periodicity of the sea-level changes, and identify their tidal components.

However, the frequency-domain analysis with traditional spectral techniques assumes that the underlying processes are stationary in time, but many natural signals are non-stationary because of their irregular or time-limited features. In this case, linear analysis approaches, such as Fourier transforms, may not be practical and efficient for analyzing these signals. Therefore, non-linear analysis approaches should be adopted to study non-stationary real-world phenomena. Currently, many advanced analysis techniques, such as wavelet transforms, are widely used to study non-linear behavior of time series [5]. Wavelet transforms, which expand time series into time-frequency space, are a powerful tool for the detection of localized and quasi-periodic fluctuations. Their extensions, the XWT and WTC, are also very useful for examining the phase relationship and the common power between the two time series [30,33,34,47,48].

From an application point of view, unlike the LSSA method, the wavelet transforms accept regularly sampled continuous data as an input for efficient analysis and reliable results. Therefore an unequally sampled time series with data voids requires pre-processing before analysis with wavelet-transform algorithms. In this study, the neural-network method was used to predict the missing values in sea-level signals from the tide-gauge-sensors records (see the missing data in the time-series plots in Figure 4(a,b)). This artificial-intelligence-inspired computation algorithm can satisfactorily represent any arbitrary nonlinear function and can find useful relationships between different inputs and outputs when a sufficient and properly trained neural network is used. This method has been widely used for multidisciplinary applications, such as the prediction of the earth-rotation parameters [37], geoid modeling [49], rainfall-runoff modeling [50], prediction of the distribution of vegetation [51], testing integrated environmental models [52], and recently sea-level investigations as well [4,18,53–56]. The multi-layer feedforward backpropagation method (MLFB), which is commonly preferred for training neural networks in these applications, was used for training the algorithm in the study (e.g., [35]). The theoretical backgrounds of the employed analysis and prediction techniques in this investigation are summarized as follows.

### 3.1. Least Squares Spectral Analysis Technique (LSSA)

In LSSA, an observed time series is considered to be a function of time *t_i_* and is represented by *f* = *f* (*t*) = {*f_i_*}, *i* = 1, 2,…, *n*. Detecting periodic signals in *f*, especially in the presence of both random and systematic noise, is the main objective of LSSA. To this end, *f* can be modeled with function *g* as follows:

(1) g=Φx

where Φ is a matrix of known base functions and *x* is a vector of unknown parameters. Here, the time series are not required to have an equal sampling rate. However, the observations *f_i_* are assumed to possess a fully populated covariance matrix C*_f_*. To estimate the model parameters *x*, the standard least-squares method (e.g., [57]) is used, in which the difference between *g* and *f* is minimized in the least squares sense. The estimate of the model parameters can be obtained as follows:

(2) $$\hat{x}={\left(\Phi^{T}C_{f}^{-1}\Phi \right)}^{-1}\Phi^{T}C_{f}^{-1}f$$

(3) $$\hat{g}=\Phi \;\hat{x}=\Phi {\left(\Phi^{T}C_{f}^{-1}\Phi \right)}^{-1}\Phi^{T}C_{f}^{-1}f$$

In the least-squares method, the model parameters are determined to minimize the difference between *ĝ* and *f*. Using the standard least squares [58], the following is obtained:

(4) $$\hat{v}=f-\hat{g}=f-\Phi {\left(\Phi^{T}C_{f}^{-1}\Phi \right)}^{-1}\Phi^{T}C_{f}^{-1}f$$

In the projection theorem, *v̂* ⊥ *ĝ*, meaning that *f* has been decomposed into a signal *ĝ* and noise *v̂* (residuals). Thus, to describe how *ĝ* represents *f*, a fractional measure *s* as the ratio of the length of this orthogonal projection to the length of *f* is used:

(5) $$s=\frac{f^{T}C_{f}^{-1}\hat{g}}{f^{T}C_{f}^{-1}f}$$

In spectral analysis, the hidden periodicities, which are expressed in terms of cosine and sine base functions, are inspected. Therefore, if a set of spectral frequencies (*ω_i_*, *i* = 1, 2,…, *m*) are specified, then the signals can be expressed as:

(6) $$\hat{g}(\omega_{i})={\hat{x}}_{1i}\;\;\;\text{cos}\;\omega_{i}t+{\hat{x}}_{2i}\;\;\;\text{sin}\omega_{i}t$$

Let *x̂* = [*x̂*_1_*_i_*, *x̂*_2_*_i_*]*^T^* and Φ = [cos *ω_i_t*, sin *ω_i_t*]. *x̂* can then be determined with the Equation (2). For different frequencies *ω_i_*, *i* = 1, 2,…, *m*, different spectral values are obtained. The least squares spectrum is then expressed as:

(7) $$s\left(\omega_{i}\right)=\frac{f^{T}C_{f}^{-1}\hat{g}\left(\omega_{i}\right)}{f^{T}C_{f}^{-1}f}\;\;\;\;\;\;,\;\;\;\;\;\;i\;=\;1,2,...,\;m$$

Equation (7) describes the least-squares spectrum. Obviously, the least-squares spectrum of *f* is the collection of the spectral values for all desired frequencies *ω_i_*, *i* = 1, 2,…, *m*. The greater the spectral value at a frequency *ω_i_*, the more powerful *f* is at this frequency [11,16,17,59]. Given Equation (7), statistically significant spectral peaks satisfy the following inequality:

(8) $$s\left(\omega_{i}\right)\geq {\left[1+\frac{v}{2}F_{v,2,\alpha }\right]}^{-1}$$

It is obvious from Equation (8) that the least-squares spectrum follows the Fisher distribution with *v* degrees of freedom and *α* level of significance [16].

In summary, the observed time series may include trigonometric base functions (see Equation (6)) to describe the periodic components of the series, along with random-walk and auto-regressive components. When the calculation of the least-squares spectrum is carried out, there will be a simultaneous least-squares solution for the parameters of the process. This approach is represented as a rigorous approach to the problem of hidden periodicities, where the parameters of the assumed linear system driven by noise are determined simultaneously with the amplitudes and phase of the periodic components and with other parameters that describe systematic noise [16,60,61].

**Figure 6.**
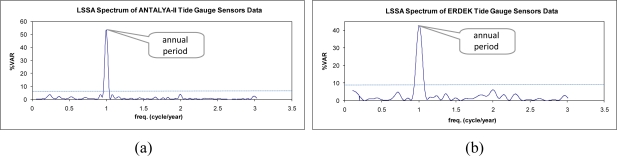

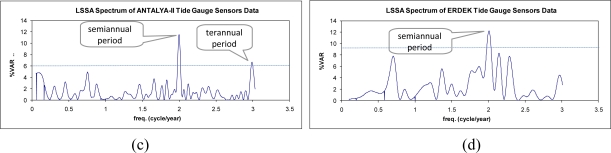
**(a,b)** LSSA spectra of the sea-level observations and **(c,d)** LSSA spectra of the observations after the removal of the signal with the highest period.

The sea-level observations were analyzed using LSSA, and the hidden periodicities of the sea-level changes in the investigated span were clarified. The periods, frequencies (cycle/year), amplitudes and phases with their root-mean square-errors and percentage variance levels (%var: a ratio indicating how much of the signal *ĝ* is contained in the observed time series *f*, see Equation (7)) are outputs of LSSA. The findings from the analysis of the sea-level data are summarized in Table 2. In the results, the annual, semiannual and terannual periodic signals were revealed in the sea-level variations recorded at Antalya-II. As recognized in the graphs in Figure 4(b,d) and the correlogram in Figure 5(b), the sea-level data recorded at Erdek is relatively noisy and has a short span. In the LSSA of the Erdek data, annual and semiannual significant frequencies were revealed. The amplitudes of the periodic signals are higher in Antalya-II records than in those for Erdek (see Table 2). The variance levels *versus* the frequencies are graphed in Figure 6, where the significance level (thin dashed line) and the significant periods are indicated. The annual periods of the sea-level changes in Antalya-II and Erdek are shown in Figure 6(a,b), and the higher-frequency signals, which were clarified by suppressing the signal with a 12-month period in the analysis, are shown in Figure 6(c,d).

**Figure 7.**
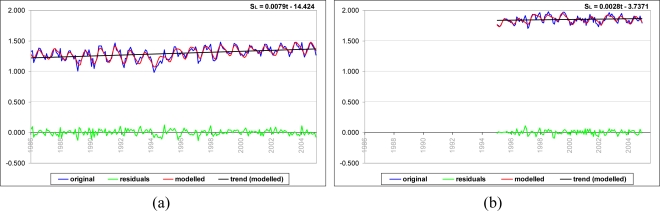
Original sea-level data (*f*) *vs*. the modeled time series after LSSA (*g*) with the trend (modeled) and the residuals (*v*) for the **(a)** Antalya-II and **(b)** Erdek tide gauges.

Figure 7 compares the modeled time series after LSSA to the original observations and shows the trend of the sea-level variations in addition to the revealed residuals in the LSSA results for Antalya-II and Erdek.

### 3.2. Neural-Network Method for Sea-Level Data Predictions

**Figure 8.**
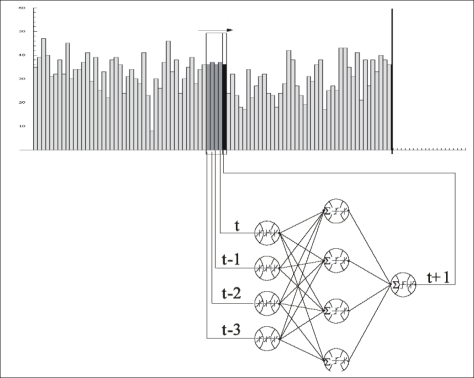
Example of neural network applications to time series predictions (e.g., using a (4-4-1)-multi-layer with four input neurons for observations *x*(*t*), *x*(*t* − 1), *x*(*t* − 2), *x*(*t* − 3), four hidden neurons, one output neuron for *x*(*t* + 1), and three layers of 20 trainable weights) [38].

The neural-network method, based on learning events using available samples *x*(*t*) and thus generating proper responses to new samples *y*(*t*), is widely used in time-series predictions, most often as feedforward backpropagation networks that employ a sliding window over the input sequence (see Figure 8). The time series prediction of closer *y*(*t*) and further *y*(*t* + *d*) values from the *n* time steps back from time *t* and using neural networks is formally depicted as:

(9a) $$\begin{array}{l} y\left(t\right)=F\left(x\left(t-1\right),\;x\left(t-2\right),...,\;x\left(t-n\right)\right)\\ y\left(t+d\right)=F\left(x\left(t\right),\;x\left(t-1\right),\;x\left(t-2\right),...,\;x\left(t-n\right)\right) \end{array}$$

where *d* is the horizon of prediction. The prediction in a time series with known period *T* is as

(9b) $$\begin{array}{l} y\left(t\right)=F\left(x\left(t-T\right),\;x\left(t-2T\right),...,\;x\left(t-\mathrm{nT}\right)\right)\\ y\left(t+\mathrm{dT}\right)=F\left(x\left(t\right),\;x\left(t-T\right),\;x\left(t-2T\right),...,\;x\left(t-\mathrm{nT}\right)\right) \end{array}$$

In the heuristic algorithm of this method, the basic element of a neural network is a processing node (Figure 8), and each processing node receives and sums a set of weighted input values and passes the summation value through an activation (transfer) function providing the output value of the node, which in turn forms one of the inputs to a processing node in the next layer of the neural network. Although transfer functions are used to decrease the number of iterations, they introduce nonlinearity into the network [49]. Thus, they increase the performance of the network. A tangent sigmoid function (Equation (10)) is one of the most frequently used transfer functions in the literature (see also Table 3):

(10) $$f(\mathrm{net})=\mathrm{tansig}\left(\mathrm{net}\right)=\frac{2}{\left(1+e^{-2(\mathrm{net})}\right)}-1$$

where *net* is the summation of the weighted input values to the processing node.

The processing nodes constitute a set of fully interconnected layers, except that there are no interconnections between nodes within the same layer in the standard feed-forward back-propagation algorithm. The structure of a typical MLFB-NN includes three types of layers: input, hidden and output (as seen in Figure 8). The input layer introduces the data for each group to the neural network. The output layer is the final processing layer that provides the output value. The hidden layers between the input and output layers, of which there may be only one, perform the basic calculations [36,49]. Each connection between the nodes has an associated weight, which is usually chosen randomly at the beginning of the training process. A value passes through an inter-connection and is multiplied by the associated weight of the connection [62].

The output of the model (y) with a single hidden and output neuron can be represented by:

(11) $$y=f(\sum w_{j,k}\;f\left(\sum w_{i,j}x_{i}\right))$$

where *w* is the weight between the layers, *x* is the input and *f* is the transfer function.

A learning algorithm is the most critical part of a neural-network method. Among a number of learning strategies, the feed-forward back-propagation learning algorithm, introduced by [63], is popular. Iterative gradient-descent and Levenberg-Marquardt training procedures are the most commonly used methods in this algorithm (in this study, the Levenberg-Marquardt (LM) training procedure was used: see Table 3 for the adopted data-prediction parameters for this investigation). The backpropagation algorithm is applied in two stages: (*i*) the network weights are randomly initialized, and the input data are presented to the network and propagated forward to estimate the output value for each training pattern set in the first stage, (*ii*) the difference (*i.e*., error E=output-observation) between the observation and the NN-output is fed backward through the network, and the weights associated with the nodes are changed in such a way that the differences between the actual and the desired outputs is minimized, in the second stage. The process is continued until achieving a minimal error or one lower than a given threshold value.

When training with the LM method, the increment of weights Δ*w* can be obtained as follows:

(12) $$\Delta w={\left[J^{T}J+\mu I\right]}^{-1}J^{T}E$$

where *J* is the Jacobian matrix and *μ* is the learning rate that is to be updated using *β* depending on the output. In particular, *μ* is multiplied by the decay rate *β* (0 < *β* < 1) whenever the performance function MSE decreases, whereas *μ* is divided by *β* whenever MSE increases in a new iteration step [64].

The performance of the neural-network model is evaluated in terms of the correlation coefficient *R* and the root-mean-square error *RMSE*, computed as:

(13) $$R=\frac{\sum_{i=1}^{N}\left(x_{i}-\bar{x}\right)\left(y_{i}-\bar{y}\right)}{\sqrt{\sum_{i=1}^{N}{\left(x_{i}-\bar{x}\right)}^{2}\sum_{i=1}^{N}{\left(y_{i}-\bar{y}\right)}^{2}}}$$

(14) $$\mathrm{RMSE}=\sqrt{\mathrm{MSE}}=\sqrt{\frac{\sum_{i=1}^{N}{\left(y_{i}-x_{i}\right)}^{2}}{N}}$$

where *x_i_* is the observation, *y_i_* is the NN output, *N* is the number of samples, *x̄* is the mean value of the observations and *ȳ* is the mean value of the outputs.

**Figure 9.**
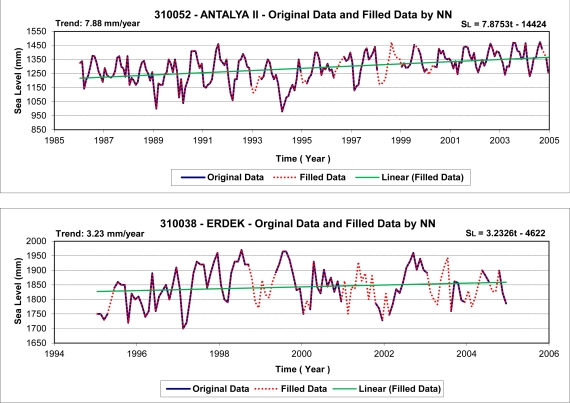
Time series of Antalya-II and Erdek tide gauges by fill by MLFB-NN method.

**Figure 10.**
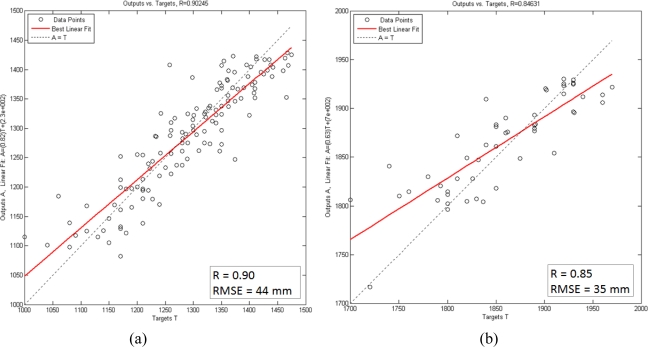
Scatter plots of target and output data: the correlations between the observations and NN outputs for the **(a)** Antalya-II and **(b)** Erdek tide gauges.

Prior to the wavelet analysis of sea-level data, the missing data in the time series (see Figure 4(a,b)) were predicted using the MLFB-NN algorithm to obtain more reliable analysis results. In the study, three-layer feedforward networks with a hyperbolic-tangent sigmoid transfer function in the hidden and output layers were employed. The prediction results are satisfactory with correlation coefficients of 0.85 and 0.90, and root-mean-square errors of 35 mm and 44 mm, for the Erdek and Antalya-II data. Figure 9 shows the filled time series of the tide gauges and its linear trend. The scatter plots of the correlations between the target (observations) and MLFB-NN outputs for Antalya-II and Erdek tide gauges are given in Figure 10.

### 3.3. Wavelet Analysis

Wavelet analysis involves a transform from a one-dimensional time series to a diffuse two-dimensional time-frequency image for detecting localized and quasi-periodic fluctuations using the limited time span of the data [3,5,26,29,30,34]. In this study, we applied CWT, and this wavelet transform is successful in clarifying high-power regions in a time series. Particularly, in some cases it is desirable to examine together two time series that are expected to be linked in some way and in such cases it has also advantage of deciding whether regions in time-frequency space with large common power have a consistent phase relationship. However, the CWT has edge artifacts because the wavelet is not completely localized in time. Therefore, the introduction of a cone of influence (COI) is suggested in which the transform suffers from these edge effects. The COI is defined so that the wavelet power for a discontinuity at the edges decreases by a factor e^−2^ and ensures that the edge effects are negligible beyond this point [5,26,30].

**Figure 11.**
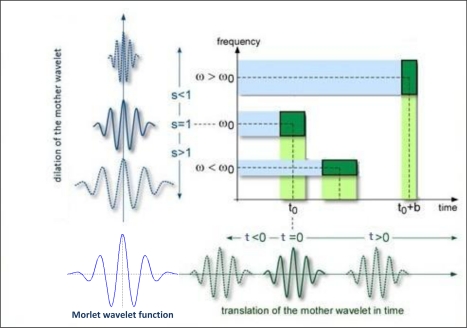
Morlet wavelet function, depending on the changes in translation (*t*) and dilation (*s-scale*) parameters [66].

The CWT of a time series is its convolution with the local basis functions, or wavelets, which can be stretched and translated with flexible resolution in both frequency and time. The CWT of the time series *X(t)* with respect to the wavelet *ψ* is defined as:

(15) $$W_{X,\psi }\left(s,t\right)=\left(X\left(t\right)*\psi_{0}\left(s,t\right)\right)$$

where *t* is time and *ψ* is the wavelet at the scale *s* (which is linearly related to the characteristic period of the wavelet). The wavelet power is defined as |*W_X,ψ_*|^2^. The complex argument of *W_X,ψ_* can be interpreted as the local phase [30]. One particular wavelet, the Morlet, is defined as:

(16) $$\psi_{0}\left(\eta \right)=\pi^{-1/4}e^{i\omega_{0}\eta }e^{-\frac{1}{2}\eta^{2}}$$

where *ω*_0_ is the dimensionless frequency and *η* is the dimensionless time. In this study, we employed the Morlet wavelet (with *ω*_0_ = 6) (see Figure 11) because it is quite well localized in both time and frequency space [5,30]. The statistical significance of CWT power was estimated against a red-noise model [26]. For other wavelet functions, [26], [29] and [65] can be referred.

The XWT spectrum of two time series (X and Y) with wavelet transforms (W_X_ and W_Y_) for the analysis of the covariance of two time series is defined by [26] as:

(17) $$W_{\mathrm{XY}}\;\left(s,t\right)=W_{X}\;\left(s,t\right)W_{Y}^{*}\;\left(s,t\right)$$

where the asterisk denotes complex conjugation. Furthermore, the power is defined as |*W_XY_*(*s,t*)|. The phase angle of *W_XY_* (its complex argument, arg(*W_XY_*)), describes the phase relationship between X and Y in time-frequency space. The statistical significance is estimated against a red-noise model [26,29,30].

The WTC is a measure of the intensity of the covariance of the two series in time-frequency space, unlike the XWT power, which reveals areas with high common power. The WTC of two time series is defined by [27] as:

(18) $$R^{2}\;\left(s,t\right)=\frac{{\left|S\left(s^{-1}W_{\mathrm{XY}}\;\left(s,t\right)\right)\right|}^{2}}{S\left(s^{-1}{\left|W_{X}\;\left(s,t\right)\right|}^{2}\right)\;S\left(s^{-1}{\left|W_{Y}\;\left(s,t\right)\right|}^{2}\right)}$$

where *S* is a smoothing operator, which is essential in coherence analysis. Otherwise, the ratio *R*^2^(*s*,*t*) would be equal to one. Values derived using the WTC vary between 0 and 1.The closer the WTC is to 1, the more coherencies there are between the time series [26]. The smoothing operator *S* is defined as:

(19) $$S\left(W\right)=S_{\mathrm{scale}}\left(S_{\mathrm{time}}\left(W\left(s,t\right)\right)\right)$$

where *S_scale_* is the smoothing along the wavelet-scale axis and *S_time_* is the smoothing in time. For the Morlet wavelet, a suitable smoothing operator is given as [30]:

(20a) Stime(W)|s=(W(t,s)*c1e−t22s2)|s

(20b) $${S_{\mathrm{scale}}\left(W\right)|}_{t}={\left(W\left(t,s\right)*c_{2}\prod \left(0.6s\right)\right)|}_{t}$$

where *c_1_* and *c_2_* are normalization constants and is the rectangle function. The factor of 0.6 is the empirically determined scale decorrelation length for the Morlet wavelet [26]. In this study, the Monte-Carlo method with red noise was used to determine the 5% statistical significance level of the coherence.

**Figure 12.**
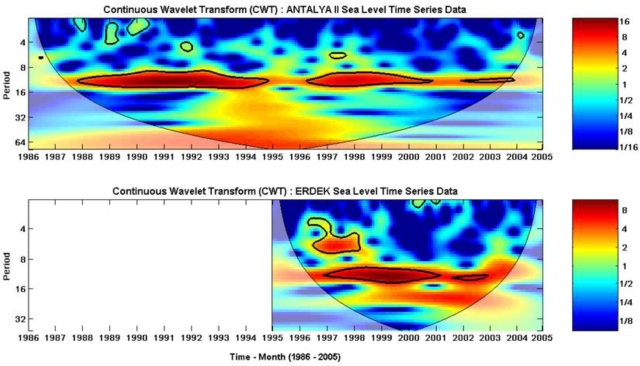
CWT power spectra of the monthly sea-level observations at the Antalya-II and Erdek tide gauges. The thick black contours indicate the 95% confidence level, and the region below the thin solid line indicates the cone of influence (COI), beyond which edge effects may distort the picture.

The time-series data filled by the NN prediction (see in Figure 9) were analyzed using wavelet transform techniques. The CWTs of the sea-level variations recorded at the Antalya-II and Erdek tide gauges are displayed in Figure 12, which show that both time series present a large scale periodicity (12 months, annual cycle) with high power and a confidence level above 95%. The smaller scale periodicities (6 months, semiannual and 4 months, terannual) are also recognized as high-power regions with the stated confidence level. The clarified periodicities in the CWT results verify the LSSA results.

**Figure 13.**
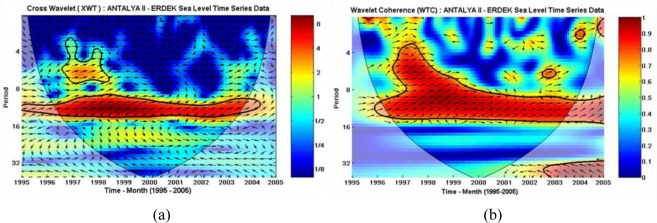
**(a)** XWT of the monthly sea-level observations at the Antalya-II and Erdek tide gauges. **(b)** WTC of the monthly sea-level observations recorded at the Erdek and Antalya-II tide gauges. In both plots, the thick black contours indicate the 95% confidence level and the region below the thin line indicates the COI.

The XWT of the two time series, Antalya-II and Erdek, is displayed in Figure 13(a), in which the areas with the high common spectral power of the time series, located at the annual cycle periodic belt in full span and partially at the semiannual cycle, are clear. In the figure, the relative phase relationships are shown as arrows (with in-phase pointing right, anti-phase pointing left). According to the plot, the sea-level changes recorded at the Antalya-II tide gauge lead the sea-level changes recorded at the Erdek tide gauge by a 20° up-pointing arrow (nearly in-phase).

Similar to that exploited by the XWT, an alternate way of investigating the phase difference of sea-level variations between the two tide-gauge records was explored through WTC. Regarding applications, whereas the XWT power reveals the areas with high common power of CWTs of two time series, the WTC can show the degree of coherence of the XWT in the time-frequency space. The WTC of the sea-level data sets is shown in Figure 13(b). The results obtained from WTC confirm the results given by XWT, but WTC was more suitable for finding coherent oscillations of the two time series than was XWT.

## 4. Conclusions

In this study, we applied LSSA and various wavelet-transform techniques, namely CWT, XWT and WTC, to time-frequency analyses of monthly sea-level variations recorded at the Antalya-II (36.8°N, 30.6°E) and Erdek (40.4°N, 27.8°E) tide gauges of TUSELS. The LSSA results clarify the amplitudes, phases, and percentage variance levels of the hidden periodicities. In the LSSA results, the 19-year sea-level observations at Antalya-II reveal significant annual (period of T = 12 month with 8.9 ± 0.4 cm amplitude), semiannual (period of T = 6 month with 2.4 ± 0.4 cm amplitude) and terannual (period of T = 4 month with 1.8 ± 0.4 cm amplitudes) cycles. The spectral analysis of the 10 year-tide gauge records at Erdek shows that the sea-level variations have significant annual (with an amplitude of 5.0 ± 0.5 cm) and semiannual cycles (with an amplitude of 1.9 ± 0.5 cm). The relative mean sea-level changes at Antalya-II and Erdek are found 7.9 ± 1.1 mm/yr and 2.8 ± 0.9 mm/yr, respectively, from the LSSA. Whereas the trend calculated for Antalya-II confirms the harmonic-analysis results of GCM reported by [42], different results were found for the Erdek tide gauge. The cause of this inconsistency between the results is the relatively short data span of the Erdek data used in this investigation.

The neural-network method was used to preprocess the sea-level data sets, and the missing parts in the time series were predicted with a feed-forward back-propagation algorithm. In the end, the quality of the prediction, as evaluated *versus* actual sea-level observations, is characterized by a correlation coefficient of the order R = 0.85 − 0.90 and a root-mean-square errors of RMSE = 35 mm − 44 mm for the time series of Erdek and Antalya-II. Considering these satisfying results, even for the relatively short Erdek sea-level data (R = 0.85, RMSE = 35 mm), we report that the MLFB-NN method is successful and useful in the prediction of the time series.

The time series preprocessed with the neural network were analyzed with wavelet transforms to observe the localized intermittent periodicities as high-power regions in the spectra with CWT by expanding the time series into time-frequency space and to inspect the common power and relative phase of the two time series in time-frequency space using XWT. We also used WTC between two CWTs to find significant coherence in the parts having low common power between the time series. The CWTs of the sea-level data sets reveal annual, semiannual and terannual periodic cycles for Antalya-II and Erdek. In the CWT images, the large-scale periodicities (annual cycles) are recognized as the full data span, whereas the smaller-scale oscillations (semiannual and terannual cycles) are partly along the spectra. The results from the CWTs of the sea-level variations confirm the LSSA findings.

The XWT of the two CWTs shows that the Antalya-II and Erdek time series has a high common spectral power at the annual-cycle periodic belt in full span and partly at the semiannual cycle. Considering the relative phase relationships derived from the XWT, the sea-level changes recorded at the Antalya-II tide gauge lead the sea-level changes recorded at the Erdek tide gauge by 20° pointing straight-up arrow (nearly in-phase). These results on the coherence of the Antalya-II and Erdek sea-level variations were confirmed and strengthened by the WTC results.

In the results of this study, we see that the LSSA has strong features in the frequency-domain analysis of the time series, especially in evaluating unequally spaced data with gaps, spikes, datum shifts and trends, such as sea-level observations. However, when series preprocessing is required for analysis in other methods (such as the wavelet-transform methods here) the neural-network method works well for predictions. As a principle advantage of the neural-network method that it is capable of approximating any continuous function, so adopting a hypothesis about the underlying structure is not required [67]. Therefore, the prediction of the time series using neural networks does not corrupt or obliterate the useful information hidden in the series. This method can provide satisfying results even for the prediction of relatively short time series. In the time-frequency analysis of the series and inspection of the coherence between two time series, the wavelet tools CWT, XWT and WTC are very useful and practical. In terms of the comprehensive and reliable investigation of the time series with quality and reliability measures of their results, each analysis method introduced in this study is suggested for analyzing serial sensors data to understand the non-stationary changes in nature. However, the availability of sufficiently long, dense and continuous time-series data in analysis would provide more efficient results.

## Figures and Tables

**Table 1.** Specifications of Antalya-II and Erdek tide gauges [2].

**Specification**	**Tide Gauges**	

Station name	**Antalya-II**	**Erdek**
	
Location (latitude, longitude)	36°50′N, 30°37′E	40°23′N, 27°51′E
PSMLS country/station code	310/052	310/038
Spanning of the used data	1986–2005	1995–2005
Acoustic gauge sensor	Aquatrak 4100	Aquatrak 4100
New acoustic systems installation year	1998	1999

**Table 2.** The LSSA results of the sea-level data of Antalya-II and Erdek tide gauges.

**DESCRIPTION ANTALYA II**	**Name**	**PERIOD (year)**	**AMPLITUDE (m)**	**SIGMA (m)**	**PHASE (DEG)**	**SIGMA (DEG)**	**SIGNIF 99%**
Periodic constituent	ANNUAL	1.000	0.089	0.004	95.853	0.255	YES
Periodic constituent	SEMI-ANNUAL	0.500	0.024	0.004	326.282	0.249	YES
Periodic constituent	TER-ANNUAL	0.333	0.018	0.004	358.778	0.250	YES

**DESCRIPTION ERDEK**	**Name**	**PERIOD (year)**	**AMPLITUDE (m)**	**SIGMA (m)**	**PHASE (DEG)**	**SIGMA (DEG)**	**SIGNIF 99%**
Periodic constituent	ANNUAL	1.000	0.050	0.005	113.433	0.286	YES
Periodic constituent	SEMI-ANNUAL	0.500	0.019	0.005	245.112	0.284	YES
Periodic constituent	TER-ANNUAL	-	-	-	-	-	-

**Table 3.** Summary of the adopted parameters in the NN prediction of the sea-level time series.

**Matlab function**	**: *newff***	Feed-forward backpropagation network
**Network type**	**: *feed-forward***	Each layer only receives inputs from previous layers
**Learning method**	**: *supervised (trainlm)***	Changes in a network’s weights and biases are due to the intervention of Levenberg-Marquardt algorithm
**Learning algorithm**	**: *backpropagation***	weights and biases are adjusted by error-derivative vectors backpropagated through the network
**Transfer function**	**: *tansig***	Function that maps a neuron’s (or layer’s) net output ***n*** to its actual output *a*. Hyperbolic tangent sigmoid transfer function 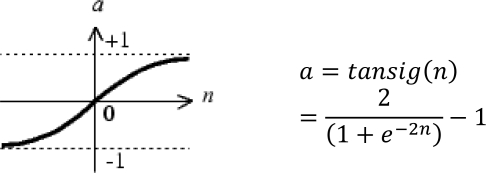
**Performance function**	**: *mse***	Mean Square Error (MSE=E^T^E/N, RMSE=sqrt(MSE))
